# WHISPER: A Location Privacy-Preserving Scheme Using Transmission Range Changing for Internet of Vehicles

**DOI:** 10.3390/s21072443

**Published:** 2021-04-01

**Authors:** Messaoud Babaghayou, Nabila Labraoui, Ado Adamou Abba Ari, Mohamed Amine Ferrag, Leandros Maglaras, Helge Janicke

**Affiliations:** 1 STIC Lab, University of Abou Bekr Belkaid, Chetouane Tlemcen 13000, Algeria; nabila.labraoui@mail.univ-tlemcen.dz; 2 DAVID Lab, Faculty of Sciences, University of Versailles Saint-Quentin-en-Yvelines, 45 Avenue des États-Unis, CEDEX, 78035 Versailles, France; adoadamou.abbaari@gmail.com; 3 LaRI Lab, University of Maroua, Maroua P.O. Box 814, Cameroon; 4 Department of Computer Science, Guelma University, Guelma 24000, Algeria; ferrag.mohamedamine@univ-guelma.dz; 5 School of Computer Science and Informatics, De Montfort University, Leicester LE1 9BH, UK; 6 Cyber Security Cooperative Research Centre (CSCRC), Perth 6027, Australia; helge.janicke@cybersecuritycrc.org.au

**Keywords:** location privacy, pseudonym change strategy, transmission range adjustment, iov privacy, iov safety, vanet

## Abstract

Internet of Vehicles (IoV) has the potential to enhance road-safety with environment sensing features provided by embedded devices and sensors. This benignant feature also raises privacy issues as vehicles announce their fine-grained whereabouts mainly for safety requirements, adversaries can leverage this to track and identify users. Various privacy-preserving schemes have been designed and evaluated, for example, mix-zone, encryption, group forming, and silent-period-based techniques. However, they all suffer inherent limitations. In this paper, we review these limitations and propose WHISPER, a safety-aware location privacy-preserving scheme that adjusts the transmission range of vehicles in order to prevent continuous location monitoring. We detail the set of protocols used by WHISPER, then we compare it against other privacy-preserving schemes. The results show that WHISPER outperformed the other schemes by providing better location privacy levels while still fulfilling road-safety requirements.

## 1. Introduction

**Figure 1:**
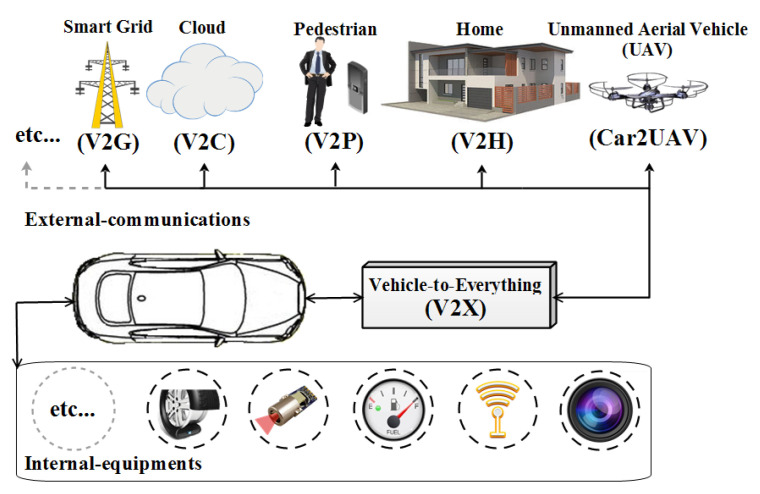
Vehicle to Everything (V2X) technology illustration.

**Figure 2:**
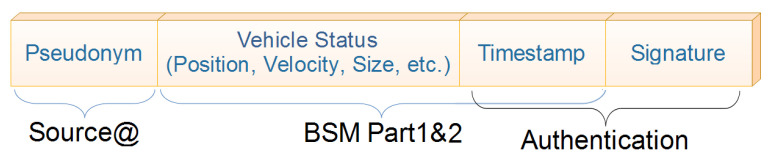
Basic Safety Message (BSM) beacon format.

A Vehicular Ad-hoc Network (VANET) with its variety of protocols (e.g., IEEE 802.11P, IEEE 1609) [1] and communication types like Vehicle to Vehicle (V2V) and Vehicle to Infrastructure (V2I) [2] has served as a basis for the promising Internet of Vehicles (IoV) paradigm [3,4,5]. IoV benefits from VANET to extend the usability range by allowing non-conventional communications and applications, e.g., Vehicle to Everything (V2X) communications, to emerge. IoV is an important sub-domain of IoT as well as a clear example of System of Systems domain [6]. Figure 1 shows V2X external communications and internal equipments. A vehicle using V2X can enhance road-safety by broadcasting a Basic Safety Message (BSM) [7,8] beacon message with a 300-m range and a frequency of 1 to 10 BSMs per second from its OBU [9,10,11]. The data included in BSMs are illustrated in Figure 2. This allows receiving vehicles to be aware of the potential dangers posed by nearby vehicles in addition to managing road-congestion, which is considered a high-level challenge [5] through the network of Road-Side-Units (RSUs).

Since BSMs contain fine-grained location data, even though they are useful for road safety, they do open privacy-related issues: Any entity with eavesdropping capability can monitor the whereabouts of IoV users. Smart cars’ safety and infotainment applications may also reveal user private information. Using these data, a system that is ultimately designed to offer safety and comfort applications to drivers can be abused by third parties, such as employers, insurance companies, or criminal organizations to track individuals [12]. The introduction of mechanisms that can preserve location privacy has become a new research trend that has attracted widespread attention among researchers. Most existing location privacy schemes, e.g., mix-zone, synchronized schemes, etc., are ineffective in achieving a high-level of privacy because of the very precise locations included in BSMs and because of their resource and overhead-consuming characteristics. The better candidate mechanism used is that of the silent period schemes by ceasing BSMs broadcasting until emerging from another location with a new pseudo-identifier. However, the major drawback of such a technique is the sacrifice of safety for the sake of privacy [13].

As safety is a substantial requirement underpinning the introduction of V2X communication, silent period schemes have been received with reservations by the research community. Our motivation is to find a solution to allow nearby vehicles to be aware (providing safety) and reduce an adversary opportunity to employ eavesdropping attacks. The purpose of protecting user location privacy in an IoV context is related to the risk of user private information being disclosed. Location privacy is directly connected to other types of privacy. Location privacy leaks can reveal the home and work address of the driver, some visits to sensitive places, travel habits, times of absence from home, etc. The correlation of this spatio-temporal information with other data allows an adversary to come to conclusions about health habits, social contacts, religious beliefs, etc. Protecting user location privacy has many benefits both to the users and the system. First of all privacy preservation improves the performance of the IoV system and reduces users’ concerns about security and privacy. Thus IoVs can attract more users to use their functions and applications, especially those that are related to safety, promoting further innovation and development in the automobile industry. In this paper, we propose a mechanism that reduces the transmission range occasionally to just inform nearby vehicles and prevent the adversary from tracking users through BSMs. The design of a pseudonym change scheme that exploits such a transmission range adjustment feature is inspired by our previous work [9] where we studied the effect of changing the transmission range using existing strategies. The novel method that is proposed in the current article, entitled “WHISPER”, maintains road-safety since vehicles are only hidden from the tracker (occasionally) and not from close vehicles (always), which makes the use of WHISPER an advantageous feature that comes in favor of safety and privacy.

The main contributions of this paper are as follows:

- We propose a novel location privacy-preserving scheme, entitled WHISPER, that maintains privacy without sacrificing safety;
- We detail the techniques and protocols used by WHISPER for adjusting the transmission range and performing a pseudonym change;
- We compare WHISPER to well-known location privacy-preserving schemes such as cooperative pseudonym change (CPN) [14], Random Silent Period (RSP) [15], and SLOW [16] in a manhattan-grid model with various densities using location privacy and QoS as metrics in addition to a comparative table.

The remainder of this paper is organized as follows: In Section 2, we review and discuss existing techniques to address the problem of location privacy in V2X. Then, we give our proposed system model in Section 3. Next, the proposed WHISPER scheme with its techniques and protocols are presented in Section 4. After that, WHISPER performances are analyzed in Section 5. Later in Section 6, we discuss the schemes in the obtained results perspective. Finally, Section 7 concludes the paper and gives future work.

## 2. Related Work

The location privacy problem is being considered as one of the crucial parameters for the adoption of a successful IoV system. There are a number of efficient privacy preservation techniques for location based ervices (LBS), however, they are using location obfuscating [17], hiding, anonymizing, and making dummies. These techniques are explained in [18], however, they are not recommended in the context of achieving safety via BSMs broadcasting and this is because of the safety-related requirements that do not recommend risking drivers’ safety for the sake of privacy as using such techniques implies tricking the adversary alongside the nearby vehicles.

Beresford and Stajano (2003) [19] introduce the mix-group concept, defined as a group region where vehicles are mixed within that region. However, broadcast beacons that contain high precision locations still represent a problem for drivers’ privacy. Based on the mix-group concept, Freudiger et al. (2007) proposed CMIX [20], a location privacy scheme that uses symmetric key-based cryptography to ensure that beacons are not readable by the adversary. This approach uses a key shared by RSUs to encrypt BSMs. Their approach did not consider the internal attacker scenario in addition to heavy infrastructure reliance.

The silent period, a concept that was firstly introduced in the field of wireless LANs by Huang et al. (2005) [15], ceases any BSM broadcasts for a specific period of time in order to achieve a good level of privacy. The strategy works well against correlation attacks [21]. However the silent periods introduce a reduction in safety relevant data being shared within the IoV – reducing the safety properties of the system and potentially invalidating safety requirements of (“ETSI TR 103 415” [22] and “ETSI TS 103 601” [23] standards). This safety-privacy trade-off is addressed in our work on WHISPER.

Buttyán et al. (2009) proposed the SLOW strategy [16]. SLOW aims at letting vehicles choose the best moment to update their pseudonyms. This decision is based on a threshold of the vehicles speed, assuming that for low speeds the risk of crashes will be low and the vehicle is allowed to stay silent for a period of time. SLOW, does not necessarily respect the beaconing frequency of once per second at minimum [9] and this occurs in congested areas. Eckhoff et al. (2011) proposed Slotswap [24], a strategy that uses a time-slotted pool to manage pseudonyms by each vehicle. Each slot is used for a period of time with the possibility to re-use pseudonyms after reaching the last time-slot. The exchange of pseudonyms between time-slots of different vehicles is also an option of their approach.

Lu et al. (2012) leveraged the feature of social spots [25] for privacy enhancement. Social spots are places where vehicles meet more frequently and are characterized with high densities such as intersections and parking lots. An adversary will be more confused in such dense areas because the probability to successfully match the old changed pseudonym with the new one inside a set of vehicles *n* will be tied inversely with the size of *n*. In the same social context, Babaghayou et al. (2019) proposed the Extreme Points Privacy (EPP) [18] scheme. EPP exploits the feature that IoV users are generally situated in district (where they live) and since turning the vehicle’s engine results in beaconing, this gives an indication to the adversary that the user is about to leave their home, thus, the authors propose to cease beaconing until leaving the district. The probability of leaving a district under different scenarios are evaluated.

Tomandl et al. (2012) [26] investigated the effects of both mix-zones and silent periods. The work was also implemented by Emmara et al. (2016) in their privacy extension PREXT [27] under the name of Coordinated Silent Period (CSP). Emmara et al. (2015) also proposed their own privacy scheme: Context-Aware Privacy Scheme (CAPS) [28]. CAPS lets vehicles choose the best opportunity to enter a silence period and to change their pseudonyms.

Pan and Li (2013) proposed the Cooperative Pseudonym Change (CPN) [14] scheme that exploits the best opportunity to achieve a synchronous pseudonym change with the help of neighbors. This way, the adversary loses tracking since vehicles are considered as the target and are indistinguishable. Yet, vehicles are not fully indistinguishable due to the fact that they broadcast fine-grained location. This means that an attacker is still able to identify the vehicles based on their precise location.

Emmara et al. (2016) also apply the silent period mechanism [15] to propose the Random Silent Period (RSP) scheme [27]. RSP is based on entering silence for a random range of time, then, it performs the pseudonym change. The scheme’s nature is considered as a spatial mix-zone because when vehicles enter the silence period and leave it after some time this implies disappearing from a point and emerging from another point which is the same idea with spatial mix-zones.

Another scheme was proposed by Zidani et al. (2018) [29] which is the Estimation of Neighbors Position privacy scheme with an Adaptive Beaconing approach (ENeP-AB) strategy that uses the number of neighbors and the predicted positions *d* as a pseudonym change trigger. Zidani et al. had also compared ENeP-AB to some other strategies like CAPs and the mix-context enhanced.

The effect of pseudonym change is mostly beneficial to privacy, however, Schoch et al. (2006) [30] shed light on some adverse consequences of intense pseudonym changes on the overall network performances and geo-routing protocols. Their results show that high pseudonym change frequencies negatively affect the system performances. Following a different direction, Zhang et al. (2019) [7] had touched upon the problem of collisions that occur while sending BSMs with high frequency, more precisely the problems occurring on the Medium Access Control (MAC) layer. They demonstrated the issue and proposed a hybrid MAC Protocol and showed its effectiveness via analysis and simulation means.

Goudarzi and Asgari (2018) proposed a congestion control mechanisms algorithm called (NOPC) [31] that is based on the beacon transmission power control. The scheme performs well in the bandwidth usage and fairness, nevertheless, its influence was not evaluated versus the achieved location privacy. In the same area, SAB Mussa et al. (2014) [32] shed light on the challenging issues that have to be addressed in the beaconing and transmission range control in the vehicular domain but without mentioning the privacy requirement [33]. A summary of recent location privacy-preserving schemes for the Internet of Vehicles is presented in Table 1. In the same table the major advantages and drawbacks of all these methods are also presented.

Based on the methods that were analyzed in the previous paragraphs and the ones presented in Table 1, it is obvious that the reviewed schemes have serious limitations. The silent period schemes are the most promising solutions but at the cost of road-safety which does not make them welcomed by the research community. Moreover, pseudonym schemes on the other hand cannot reassure privacy preservation since an adversary can still track vehicles that are broadcasting their locations even if they change pseudonyms by performing the so-called linking attack. The proposed idea in this research paper (WHISPER) comes to fill these limitations, by ensuring privacy along with a high road-safety level, by acting as a silent period scheme on some occasions.

## 3. System Model

In this section, we define and describe the Overall System Model comprised of a network model, the threat/attacker model, a set of assumptions that are taken while making such a research study in addition to technical details and a mathematical model that reflects the fundamentals of using certificates under an IoV system.

### 3.1. Network Model

**Figure 3:**
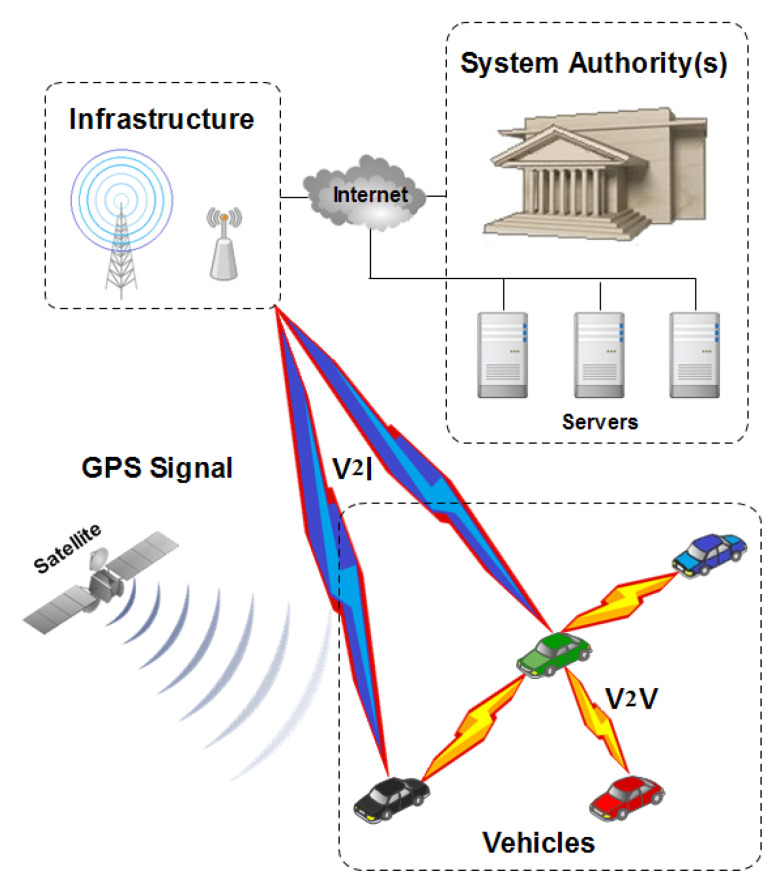
The different entities of the vehicular network.

The network model used in this paper is illustrated in Figure 3, and contains the following entities:

- Vehicles: They are the basic units of the VANET paradigm which provides a platform to the V2X applications. The communication is done via the 802.11p [3] standard and can perform Vehicle to Vehicle (V2V) and Vehicle to Infrastructure (V2I) communications. The set of vehicles is defined as $V=\{v_{1},v_{2},\cdots v_{n}\}$.
- System Authorities: They are the the entities related to the law-side (e.g., governmental bodies) that have different resources, tasks, and roles like: Distributing, issuing, revoking pseudonyms, etc. [41]. It is also important that the system authorities almost always are able to fulfill the accountability requirement in order to track down and determine misbehaving users [42].
- Infrastructure: Composed by different components and stations, its role is to relay and facilitate the connectivity between the vehicles and any potential attached network entity. The most interesting feature is the Vehicle to Infrastructure (V2I) communications. Additionally, V2X communications may exploit the infrastructure.

### 3.2. Threat Model

**Figure 4:**
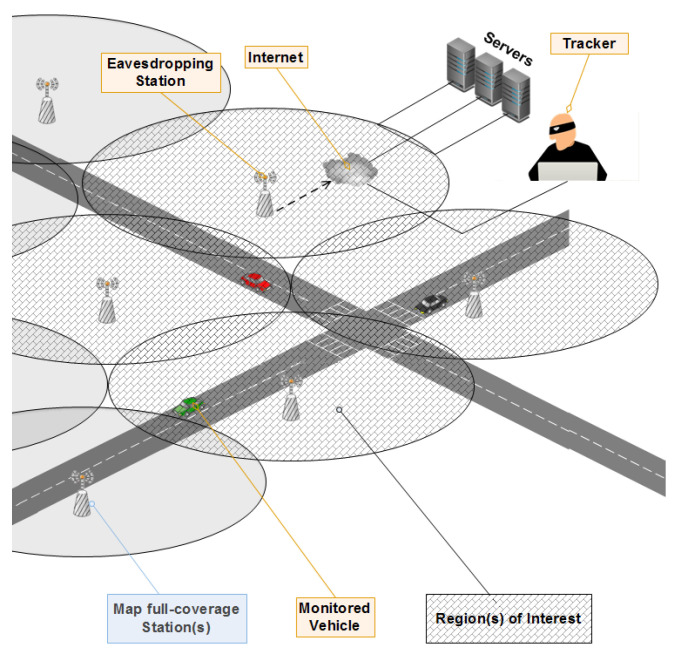
Threat model and its resources, capabilities, and coverage.

The threat model is shown in Figure 4 and is composed from the following elements:

- Tracker: The malicious element in the system, even though it is not active, can still execute many influencing attacks such as eavesdropping, tracking, profile-generation, etc. In most researches, the Global Passive Adversary (GPA) [10] is considered as the adversary type used while evaluating their own schemes. The GPA is a strong adversary that covers almost the whole map (or at least, the region of interest) and can obtain every sent message passively, i.e., no data forgery, modification, or creation is executed by him.
- Eavesdropping stations: They are stations capable of collecting the transmitted BSMs where all of the coverage mode, the emplacement, and the transmission range of vehicles do affect the amount of the collected packets.
- Tracker resources: They are the various materials and software used in conjunction with the eavesdropping stations. They can be high performance servers, tracking algorithms and methods, etc.

### 3.3. Assumptions

**Figure 5:**
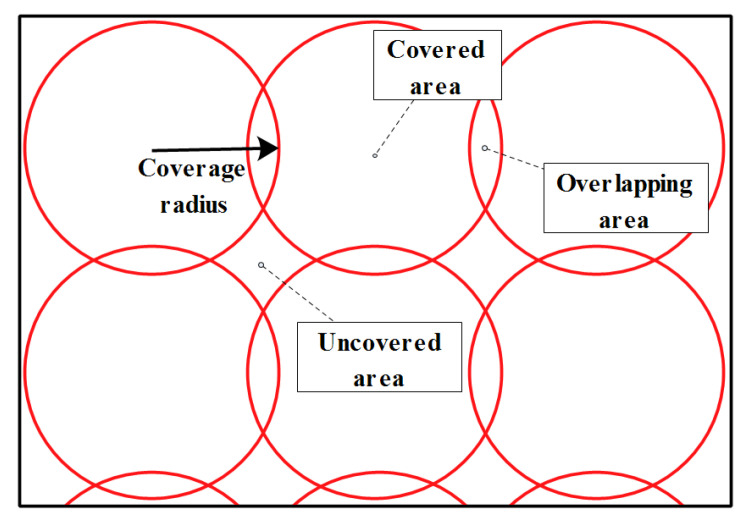
The used coverage mode (moderate mode) details.

We put a set of assumptions for what is included in this research:

- Vehicles are able to adjust their transmission range by changing the used transmission power.
- The adversary is setting eavesdropping stations in accordance to the standardization (300 m of transmission range for vehicles).
- The distributed eavesdropping stations do overlap in 30 m and have a moderate coverage mode to collect much BSMs by effectively exploiting the resources. This is illustrated in Figure 5.
- At a given time, the adversary can exclude the remaining of the map and only focuses on a region of interest. This is done at the aim of targeting only specific vehicles for better calculations and to well-exploit the resources (it is shown in Figure 5).
- Vehicles use Public Key Infrastructure (PKI) certificates mechanism to communicate, thus, changing the used pseudonym implies using a new certificate. This later is assumed to be issued from a trusted authority by doing the certificates refill request.

### 3.4. Certificates Management

Since the use of pseudonyms implies the use of certificates, a better management is envisioned in order not to affect the functioning of the whole system. With this said, having a large set of certificates with less consumption frequency would be preferred, hence minimizing their refill requests. In order to quantify the used certificates for each vehicle per unit of time, an estimation is highly needed. For that aim, we provide the following equations related to the used certificates:

- The estimated number of certificates per day $NbrCerts_{day}$ without changing the certificate by a number other than that of the expiration is calculated as in Equation (1):
  (1) NbrCerts_day=NbrCerts_m×DrivTime_day
  where NbrCerts_m is the number of used certificate per minute and DrivTime_day is the estimated amount of time (in minutes) that the user is going to drive per day.
- The number of necessary certificates per year, assuming that a normal refill is made each year, is like in Equation (2):
  (2) NbrCerts_year=NbrCerts_day×365.
- From here, the estimated remaining certificates after *d* days since the last yearly refill (NbrRemainCerts(d)) is calculated as written in Equation (3):
  (3) NbrRemainCerts(d)=NbrCerts_year−d×NbrCerts_day.
- However, certificates may also get invalid due to a certificate change (triggered by a pseudonym change for example) and thus, the exact remaining certificates after *d* days since the last yearly refill (RealNbrRemainCerts(d)) can be calculated as in Equation (4):
  (4) RealNbrRemainCerts(d)=NbrCerts_year−d×NbrCerts_day−NbrCerts_chngd
  where NbrCerts_chngd is the number of times the certificate got changed due to a reason other than a normal expiration.

## 4. The Proposed WHISPER Strategy

WHISPER uses the change of transmission power to preserve or at least augment the level of location privacy in addition to ensuring road-safety while driving. Vehicles monitor the neighborhood and their proper speeds on-the-fly in order to adjust their beacons transmission range. This is because the adversary, in our assumptions, distributes eavesdropping stations intelligently and economically according to the standardization (that vehicles transmit with 300 m of range). Thus, when driving in low speeds the vehicle (i.g., $v_{i}$) may reduce, according to the value of its speed (and the surrounding vehicles’ speeds), its own range to ensure that:

- The safety of its neighbor vehicle(s) (e.g., $v_{j}$) is preserved unlike the case of the silent period schemes that do not make much safety-considerations when going to enter silent. This is fulfilled by continuously checking its own speed. Thus, when in high speeds, the risk of a sudden crash will be high, which is why $v_{i}$ ought to be visible earlier to the surrounding vehicles ($v_{j}$).
- Its own safety. This is fulfilled by the neighbor vehicle(s) $v_{j}$ that are using the same behavior as $v_{i}$ while driving in different speeds. They aim, as a consequence, to inform $v_{i}$ earlier when they are driving in high speeds. Once it has received a BSM with a powerful transmission range, $v_{i}$ takes that as a parameter and adjusts, in its role, its own transmission range based on that parameter and on its own speed. By doing so, $v_{i}$ will be visible to the other neighbors, $v_{j}$ as well.
- The two aforementioned points lead to a collective awareness that will ensure the safety of both $v_{i}$ and its neighbor $v_{j}$.
- To benefit and exploit the already deployed eavesdropping mode, as these eavesdropping stations will not be able to collect BSMs all the time even if the vehicles are inside the area of the eavesdropping station. This is because each eavesdropping station is placed at the aim of intercepting every sent BSM in the range of 300 m.

### 4.1. System Initialization

Each vehicle $v_{i}$ is equipped with *M* certificates and each one of them is defined as ($Cert_{i,j}$) where *j* represents the *i*-th certificate of $v_{i}$. Thus, each vehicle $v_{i}$ has a set of certificates $C_{i}$ defined as follows: $C_{i}=\left\{Cert_{i,1},Cert_{i,2},\cdots Cert_{i,m}\right\}$. When referring to a pseudonym change, this implies the use of another certificate.

Before we dive into the detailed modus-operandi of WHISPER, we define the set of concepts (find them in Table 2) that are key-parameters used to determine the exact behavior of WHISPER.

Generally speaking, in WHISPER, every vehicle $v_{i}$ can be in one of the following main states:

- *Vehicle ON*: Is the state when a vehicle is turned on (to be ready for driving).
- *Listening*: Once on, $v_{i}$ keeps monitoring the transmission medium to detect any transmitted BSM. Both its neighbor(s) status (found in their transmitted BSMs) and its own speed.
- *Receiving BSMs*: When receiving a BSM from $v_{j}$, $v_{i}$ proceeds into diverse calculations at the aim of knowing the status of $v_{j}$.
- *Adjusting the transmission power*: In this status, $v_{i}$ takes as parameters its own speed and the neighbors’ speed and may, accordingly, adjust its transmission range in order to ensure road-safety and preserve location-privacy of the present vehicles.
- *Checking pseudonym change condition*: This status comes after the Beacon_Interval_Time expires. $v_{i}$ will check its eligibility for a pseudonym (and certificate) change. When favorable, $v_{i}$ moves into the next status.
- *Pseudonym change:* In this status, a pseudonym change takes place and the BSM will be sent right after.
- *Sending a BSM*: This status happens after the *Pseudonym change* action. Sometimes, the pseudonym change trigger will not be satisfied, thus, $v_{i}$ just sends the BSM. In both scenarios, $v_{i}$ returns to the next status (*Listening*) afterwards.
- *Vehicle OFF*: The status where a vehicle is turned off and thus the ending status.

**Figure 6:**
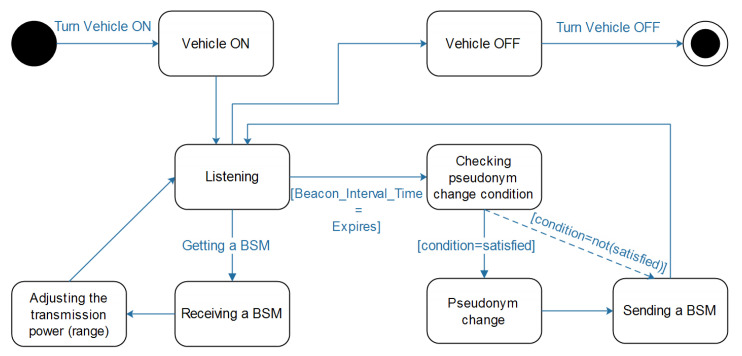
The state diagram of WHISPER.

A state diagram is presented in Figure 6 which gives a better illustration and understanding on the aforementioned states and the existing transitions.

### 4.2. Receiving Beacon Messages Protocol

**Figure 7:**
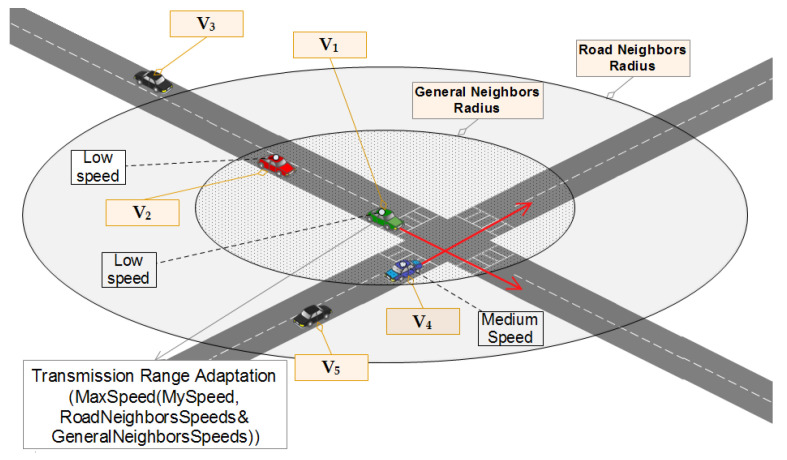
WHISPER behavior in the presence and influence of general neighbors on the transmission range adjustment.

**Figure 8:**
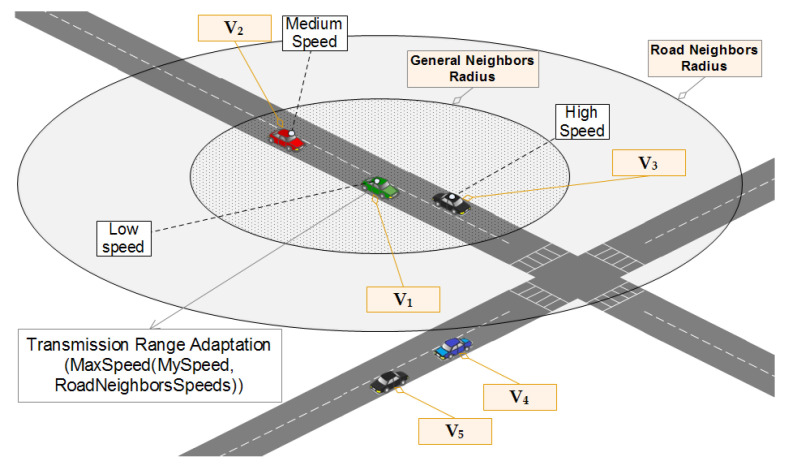
WHISPER behavior in the presence and influence of road neighbors on the transmission range adjustment.

Vehicles are always ready to receive BSMs. When receiving a BSM, the receiving vehicle $v_{i}$ considers the sender’s position and calculates the distance between itself and the sender. By doing this simple calculation, $v_{i}$ will be able to get a set of useful information that will determine its behavior. The pseudo-code of receiving a beacon message in WHISPER is illustrated in Algorithm 1. The main conclusions that $v_{i}$ is going to have after parsing the BSM sent by $v_{j}$ are the following:

- Knowing the distance between itself and $v_{j}$.
- Whether to consider $v_{j}$’s BSM for transmission power adjustment or just ignore it.
- It considers $v_{j}$’s BSM for transmission power adjustment if Dist is less than or equal to GeneralNR (shown in the scenario that is illustrated in Figure 7).
- It considers $v_{j}$’s BSM for transmission power adjustment if Dist is less than or equal to RoadNR but also share the same road segment with each other (shown in the scenario that is illustrated in Figure 8).
- It considers itself eligible for the pseudonym change if Dist is less than or equal to CloseNR. It does change Close to True as a consequence.

This protocol is called whenever $v_{i}$ receives a BSM generated by $v_{j}$ and with each call, less than 10 instructions are executed thus a linear complexity per each call O(10). With this said, by receiving (R) BSM, the complexity of the whole protocol will be as in Equation (5):

(5) O(R×10)=O(n).

This indicates that the ReceivingBeaconMessages protocol is neither time nor resources consumer.
**Algorithm 1** Receiving Beacon1:**procedure**Receiving_Beacon(beacon* bsm)2:    His_Pos←BSM.SenderPos();3:    Dist←Calc_Dist(My_Pos,His_pos);4:    **if** ((Dist<=GeneralNR)OR((Dist<=RoadNR)AND(MyRoadID=HisRoadID)) **then**5:        His_Speed←BSM.SenderSpeed();6:        Speed←Max(My_Speed,His_Speed);7:        **if** (Dist<=CloseNR) **then**8:           Close←TRUE;9:        **end if**10:    **end if**11:    Process_Beacon(BSM);12:**end procedure**

### 4.3. Transmission Range Adjustment Protocol

Each vehicle $v_{i}$, and after the Beacon_Interval_Time expires, will send a BSM to inform the nearby vehicles about its location. Particularly, WHISPER adjusts the transmission range prior to the final BSM broadcast. The adjustment is done each time a BSM is received by $v_{i}$ as explained before. When going to broadcast, $v_{i}$ uses the value of Speed to decide the appropriate transmission range (between all of the four levels: Low, Medium, beyond-Medium, and High). Algorithm 2 shows the pseudo-code of sending a BSM after making the transmission range adjustment step. Additionally, Speed is reinitialized to 0 after that and Checking_Pseudonym_Change_Trigger() is called during this protocol and that is to see the eligibility of changing $v_{i}$’s pseudonym (and certificate respectively). Moreover, Counter is decreased depending on the value of Speed and this is to trigger the pseudonym change (will be seen in the next point). However, if Speed is at max level, there will be no meaning for changing the pseudonym and that is because the attacker is able to collect every sent beacon (the maximum transmission range is used) and that is why Counter is reinitialized to its default value Def_Val.

This protocol is called whenever $v_{i}$ Beacon_Interval_Time expires and thus, one time per call. However, it calls, in its role, the Checking_Pseudonym_Change_Trigger() protocol. In total, there are 7 instructions without counting the called protocol (O(7)). With this said, the complexity of the TransmissionRangeAdjustment protocol is defined as in Equation (6):

(6) O(1×(7+O(Checking_Pseudonym_Change_Trigger())))=O(Checking_Pseudonym_Change_Trigger()).

This indicates that the TransmissionRangeAdjustment protocol does depend on the PseudonymChangeTrigger protocol.
**Algorithm 2** Sending Beacon1:**procedure**Sending_Beacon2:    **while** (OBU_Is_On) **do**3:        Wait(Beacon_Interval_Time);4:        Prepare_Beacon(BSM);5:        Speed←Max(My_Speed,Speed);6:        **if** (Speed<18) **then**7:           nic.mac80211p.txPower←0.2;8:           Counter←Counter−5;9:        **else if** (Speed<36) **then**10:           nic.mac80211p.txPower←0.8;11:           Counter←Counter−10;12:        **else if** (Speed<54) **then**13:           nic.mac80211p.txPower←3.1;14:        **else**15:           nic.mac80211p.txPower←7;16:           Counter←Def_Val;17:        **end if**18:        Speed←0;19:        Checking_Pseudonym_Change_Trigger();20:        Send_Beacon(BSM);21:    **end while**22:**end procedure**

### 4.4. Pseudonym Change Trigger Protocol

**Figure 9:**
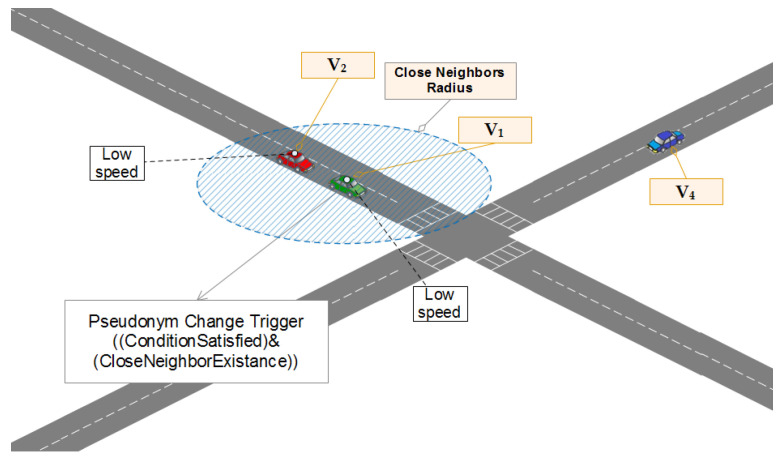
WHISPER, pseudonym change process triggered by a close neighbor’s status.

In order to avoid wasting pseudonyms (certificates) in an inappropriate opportunity, finding an almost good opportunity requires that the pseudonym change trigger must be implemented delicately. Algorithm 3 shows, in a pseudo-code, the way vehicles perform a check to see the eligibility for changing their pseudonyms. When the trigger *Counter* reaches or drops below (0) (which is an indicator that $v_{i}$ was sending BSMs with a short range for some important period of time) $v_{i}$ changes its pseudonym then initializes the trigger *Counter*. This whole process provides high confusion chances since the pseudonym change is performed not in the favor of the tracker (see the scenario illustrated in Figure 9). The PseudonymChangeTrigger protocol is used each time the TransmissionRangeAdjustment is executed. Its complexity depends on a small and fixed number of instructions (5), thus, can be defined as in Equation (7):

(7) O(5)=O(1).

The PseudonymChangeTrigger protocol has O(1) as a complexity.
**Algorithm 3** Checking Pseudonym Change Trigger1:**procedure**Checking_Pseudonym_Change_Trigger2:    **if** ((Counter<=(Def_Val/2))AND(Close)) **then**3:        Counter←Def_Val;4:        Pseudonym_Change();5:    **else if** (Counter<=0) **then**6:        Counter←Def_Val;7:        Pseudonym_Change();8:    **end if**9:    Close←FALSE;10:**end procedure**

## 5. Performance Evaluation

To validate the performances of WHISPER, we use simulation runs in a manhattan grid model created using the NETEDIT script included in SUMO; the mobility simulator [43]. SUMO is considered as one of the most credible and realistic mobility simulators. The mobility and environment information used for the simulation are presented in Table 3. The manhattan grid model consists of 9 intersected roads with attached segments where each segment has a length of 200 m.

**Figure 10:**
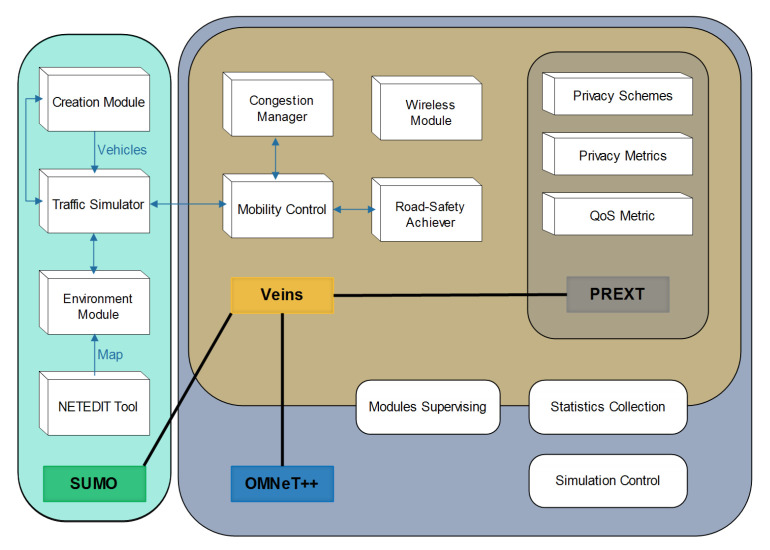
The block diagram of the different used simulation tools.

Concerning the network simulator, we use OMNeT++ [44]; the component is c++ based and discrete events simulator. OMNet++ allows the integration of diverse frameworks depending on the simulation nature like Veins [45], which is the vehicular network simulator. Veins acts as a bridge between the mobility simulator SUMO and the network simulator OMNet++. We also employ the PREXT extension [27] that is developed by Emmara et al.; a Veins extension that integrates a set of (1) location privacy schemes, (2) some privacy metrics such as the traceability and the normalized traceability (described in [46]), and (3) a Quality of Service (QoS) metric (the consumption of pseudonyms/certificates). A block diagram is elaborated in order to facilitate the comprehension of the interaction between the different simulation tools (shown in Figure 10). Based on PREXT, WHISPER is evaluated and compared against some other schemes under the same environmental condition using the aforementioned metrics. The schemes’ parameters and the evaluation metrics are also presented in Table 3.

### 5.1. The Adversary’s Achieved Traceability

**Figure 11:**
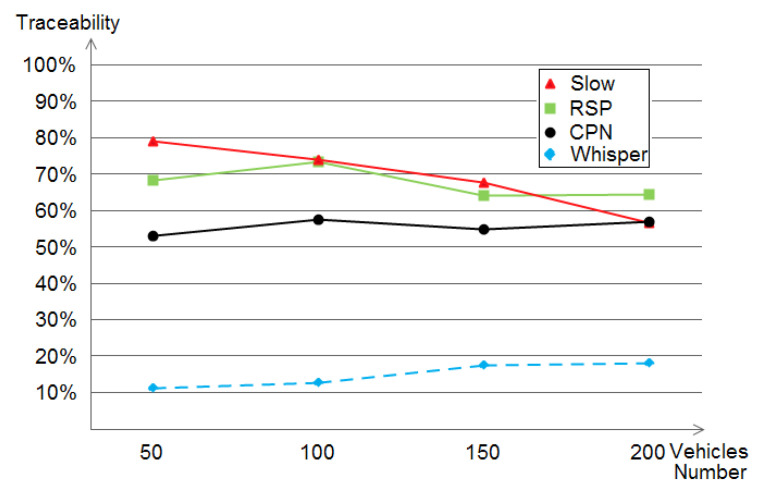
The achieved traceability by SLOW, Random Silent Period (RSP), Cooperative Pseudonym Change (CPN), and WHISPER within different densities.

Traceability, the location privacy metric used in this study, is defined as the correctness of an adversary to build the target vehicle’s traces using its eavesdropped beacons [46]. The results, provided in Figure 11 show that WHISPER outperformed SLOW, RSP, and CPN in the traceability metric with a clear difference (ranging in the interval of 10% to 20%). An important remark is that at dense situations (e.g., with the density of 200 vehicle), the traceability gets augmented a bit. The reason behind the decrease in the privacy level is due to the higher density of vehicles, which can help the attacked collect BSMs from the legitimate cars.

In general, as presented in Figure 11, WHISPER performs better in terms of the level of privacy that it offered since it achieves a traceability ranging in the interval of 10% to 20%. We interpret this as being WHISPER reducing the vehicle’s transmission range according to its and/or the neighbor vehicles’ speeds (according to the safety situation) followed by CPN, RSP, then SLOW, in addition we observe that the traceability decreases when augmenting the number of vehicles in SLOW. The reason is that, in high densities, vehicles would drive with lower speeds, thus, SLOW performs better.

### 5.2. The Adversary’s Achieved Normalized Traceability

**Figure 12:**
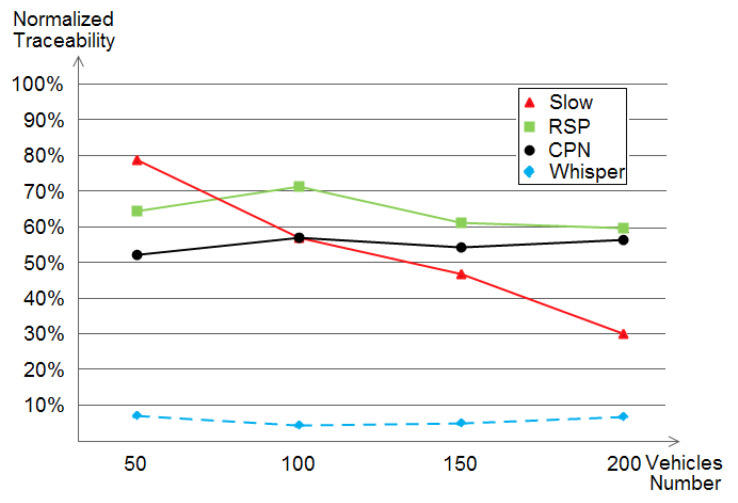
The achieved normalized traceability by SLOW, RSP, CPN, and WHISPER within different densities.

As some vehicles may not perform the pseudonym change, building their traces becomes easy, thus, excluding them gives more fairness to the real level of privacy [46]; that is the normalized traceability. With this definition, our conducted simulation under the normalized traceability aims to give a more credible and better privacy-reflecting metric to quantify the achieved privacy level of WHISPER, SLOW, RSP, and CPN (shown in Figure 12).

As stated above, by taking the case of just the vehicles which did change their pseudonyms, we get the achieved normalized traceability as shown in Figure 12. The results always give WHISPER the leading position since it outperforms the other schemes but this time by achieving an even higher privacy level represented in a lower than 10% of normalized traceability. The same order of performance remains: CPN, RSP, then SLOW. However, SLOW has achieved better-normalized traceability of about 30% due to removing vehicles that did not change their pseudonyms at all from the calculation.

### 5.3. Pseudonym Consumption

**Figure 13:**
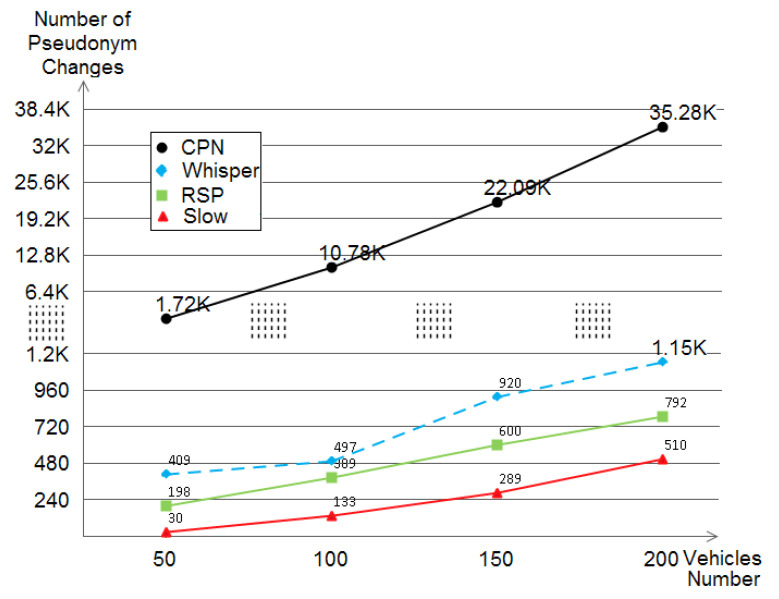
The pseudonyms changes (consumption) evaluation of CPN, WHISPER, RSP, and SLOW within different densities.

Also considered is the QoS metric. The pseudonym consumption has multiple effects and impacts like the use of different pseudonyms (thus, certificates), extra-communications with the corresponding authorities to refill pseudonyms, affecting the routing algorithms [30], etc. For this reason, the pseudonym consumption metric is crucial. With a clear view, Figure 13 shows that SLOW is the less-pseudonym consuming scheme followed by RSP and WHISPER respectively, while CPN had a considerable high pseudonyms consumption level. We argue this by the scheme’s nature, when the trigger of *k* neighbors is satisfied, a pseudonym change is performed and as *k* was taken as 2 by the default parameters, a lot of pseudonym changes occurred.

## 6. Discussion

For an overall investigation, the performances of CPN, RSP, SLOW, and WHISPER were evaluated in terms of (1) location privacy that gives WHISPER the leading in both (a) traceability and (b) normalized traceability and (2) QoS comes in the favor of SLOW. CPN, under the default parameters (i.e., k=2), has resulted in a very high pseudonym consumption, thus, considered as a non-wise choice for a deployed pseudonym scheme. The results, clearly show that WHISPER has a very good level of privacy since it achieves traceability ranging in the interval of 10% to 20%. In terms of normalized traceability, WHISPER outperformed the other schemes achieving an even higher privacy level.

Despite WHISPER consuming more pseudonyms (with a remarkably low amount in general) than SLOW and RSP, having it a very high location privacy level represented in the traceability and the normalized traceability gives it the leading position. Thus, we can say that WHISPER, as also compared and summarized in Table 4, has outperformed the other schemes especially in both the safety and location privacy that are known to be on the top of the security requirements.

Except for the evaluation comparison, WHISPER is an important solution that offers privacy preservation while maintaining at the same time road-safety. This is achieved since vehicles are only hidden from the tracker (occasionally) and not from the close vehicles (always), which makes the use of WHISPER an advantageous method that comes in favor of safety and privacy.

## 7. Conclusions and Future Work

In this paper, WHISPER, a novel location privacy-preserving scheme that is based on reducing the transmission range while sending the safety beacons was proposed. We presented WHISPER protocols, techniques, and algorithms and compared them against other methods, namely CPN, RSP, and SLOW in terms of the location privacy level (traceability, normalized traceability) and QoS (pseudonyms consumption) metrics. WHISPER clearly outperformed the other schemes in location privacy evaluation, which is an important security requirement, but consumed, lightly, more pseudonyms than SLOW and RSP as the QoS evaluation demonstrated. Furthermore, WHISPER showed its robustness during the evaluation and also provided (1) road safety that is missed by all other silent period schemes in conjunction with (2) location privacy.

The reason why WHISPER is a road-safety mechanism is that the vehicle is only hidden from the tracker (occasionally) and not from close vehicles (always) which made the use of WHISPER (or at least, the change of transmission range protocol) an advantageous feature that works in favor of safety and privacy alike.

As this new technique has not been exploited before in the privacy field, we intend on evaluating the achieved location privacy level versus an internal attacker i.e., when vehicles act as malicious eavesdropping stations in order to bypass the reduction of transmission range and increase the coverage of the attacker. Also, some of the values (e.g., existing in Algorithm 2) are set heuristically, evaluating the performance by optimally adjusting those values dynamically would certainly enhance the obtained privacy level of WHISPER. Moreover technologies like blockchain [47], cryptography [48], IDSs [49], and Edge Computing [50] which are widely recognized as key enablers for IoV could be integrated or used in parallel with our solution. Finally, using other metrics like the number of sent BSMs, the number of verified signatures, and evaluating WHISPER’s performance under different scenarios like the free-way model are some of our future plans.

## Figures and Tables

**Table 1:** Comparison of related works.

Year	Scheme	Network Model	Technique Used	Pros (+)	Cons (−)
2007	Freudiger et al. [20]	Vehicular networks	Symmetric key-based cryptography	+ Provides location privacy	− The proposed scheme did not consider the internal attacker scenario
2009	Buttyán et al. [16]	Vehicular networks	Pseudonym changing scheme	+ Ensures both silent periods and synchronized pseudonym change in time and space	− Intrusion detection is not considered
2011	Eckhoff et al. (2011) [24]	Intelligent transportation systems	A time-slotted pool to manage pseudonyms by each vehicle	+ Affordable location privacy	− Resistance against Sybil attacks is not considered
2012	Lu et al. [25]	Vehicular networks	The feature of social spots, which are places where vehicles meet more frequently	+ Provides location privacy	− Limited analysis against threat models
2013	Pan and Li [14]	Vehicular networks	Cooperative pseudonym change scheme based on the number of neighbors	+ Provides anonymity	− Intrusion detection is not considered
2017	Ferrag and Ahmim [34]	Vehicular peer-to-peer social network	Searchable encryption with vehicle proxy re-encryption	+ Provide privacy for resources, authentication and data integrity of the demand’s source	− Limited analysis with threat models against botnet attacks
2018	Zidani et al. [29]	Vehicular ad-hoc network	Estimation of neighbors position privacy scheme with an adaptive beaconing approach	+ Provides location privacy	− Limited analysis against threat models
2019	Babaghayou et al. [18]	Vehicular networks	Location-privacy evaluation within the extreme points privacy	+ Provides location privacy	− Limited analysis against threat models
2020	Aman et al. [35]	Internet of vehicles	Physical unclonable functions	+ Reduces the overhead of authentication and improves the throughput of application layer packets	− Resistance against DDoS attacks
2020	Song et al. [33]	Internet of vehicles	Fog-based identity authentication scheme	+ Reduces the burden on the traffic control center	− Resistance against Botnet attacks
2020	Sutrala et al. [36]	Internet of vehicles	Elliptic Curve Cryptography (ECC) technique	+ Secures against a passive/active adversary through various security analysis	− Communication and computation overhead
2020	Dwivedi et al. [37]	Internet of vehicles	Blockchain technology	+ Supports data immutability property	− The limited analysis against the threat models
2020	Zhang and Li [38]	Internet of vehicles	- Task allocation and data aggregation mechanism - Robin Steiner bargaining game model	+ Encourages selfish nodes to perform data transmission and reduce time delay	− Limited analysis against threat models
2020	Vasudev et al. [39]	Vehicle to Vehicle (V2V) communication in the Internet of Vehicles	Lightweight mutual authentication protocol	+ Secure communication, while minimizing computational cost	− Limited analysis against threat models
2021	Kamal et al. [39]	V2V communication in the Internet of Vehicles	Blockchain technology and channel characteristics of wireless networks in V2V communication	+ Provides real time adversary detection within the network	− Energy and computation overhead
2021	Bagga et al. [40]	Internet of Vehicles-enabled intelligent transportation system	Mutual authentication and key agreement protocol	+ Secures against a passive/active adversary through various security analysis	− Communication and computational overhead

**Table 2:** WHISPER keywords, concepts, and detailed definitions.

The Concept	Its Definition
The different speed levels (km/h)	Low (≥0 & <18), Medium (≥18 & <36), beyond-Medium (≥36 & <54), High (≥54)
My_Pos and His_Pos (x,y,z)	The position of $v_{i}$ which does the calculation and $v_{j}$ which sends the BSM
My_Speed and His_Speed (km/h)	The speed of $v_{i}$ which does the calculation and $v_{j}$ which sends the BSM
Speed (km/h)	The highest speed that was encountered while $v_{i}$ was waiting
Dist (m)	The distance between the sending vehicle $v_{j}$ and the receiving vehicle $v_{i}$
Calc_Dist(A,B) (m)	Calculates the distance between point *A* and *B*
BSM.X() (depends)	X() is the method applied on BSM to retrieve different fields like position, speed, etc.
GeneralNR (m)	A virtual range with the same value for each receiving vehicle $v_{i}$. This range determines
	whether a sending vehicle $v_{j}$ is considered as a “General Neighbor” to $v_{i}$ or not
	If $v_{j}$ is inside that range when sending its BSM, then it is considered to be $v_{i}$’s
	General Neighbor
RoadNR (m)	A virtual range with the same value for each receiving vehicle $v_{i}$. This range determines
	whether a sending vehicle $v_{j}$ is considered as a “Road Neighbor” to $v_{i}$ or not
	$v_{j}$ is only considered as a Road Neighbor to $v_{i}$ if it is inside the RoadNR range and if
	it and $v_{i}$ share the same road segment
CloseNR (m)	A virtual range with the same value for each receiving vehicle $v_{i}$. This range determines
	whether a sending vehicle $v_{j}$ is considered as a “Close Neighbor” to $v_{i}$ or not. $v_{j}$ is only
	considered as a Close Neighbor if it is inside the CloseNR range. Noting that CloseNR
	range ought to be very small in order to let both $v_{i}$ and $v_{j}$ be as much indistinguishable
	as possible to confuse the attacker when doing the pseudonym change action
Close (boolean)	A local variable that each vehicle $v_{i}$ has. Being *True* means that $v_{i}$ is currently at the
	proximity of another vehicle $v_{j}$. In the other case, when $v_{i}$ is alone (with regard to the
	CloseNR range), its value becomes *False* (to achieve road-safety, entertainment,
	congestion-aware actions, etc.)
Proccess_Beacon(BSM) (procedure)	This procedure uses the received BSM packet for the IoV objectives and requirements
	(to achieve road-safety, entertainment, congestion-aware actions, etc.)
OBU_Is_On (boolean)	A true or false value which means a sending vehicle $v_{i}$ is on or off respectively
Beacon_Interval_Time (s)	An amount of time in where $v_{i}$ is waiting before sending the next BSM
Prepare_Beacon(BSM) (Beacon)	Generates a BSM packet that will be ready for broadcasting
nic.mac80211p.txPower (milliwatt)	The transmission power given to the network interface used to control the transmission
	range of $v_{i}$
Counter (number)	A counter variable used later on to decide the pseudonym change action
Def_Val (number)	The default value of counter. It is used to both reinitialize counter and to do a test to
	find out the eligibility of $v_{i}$ for changing its pseudonym
Send_Beacon(BSM) (Beacon)	Gives the BSM packet to the lower layers which will broadcast it to the neighbors
Checking_Pseudonym_Change_Trigger()	Checking whether the trigger of $v_{i}$ for changing its pseudonym is met or not
(procedure)	
Pseudonym_Change() (procedure)	Once the conditions are met and once it is executed correctly, $v_{i}$ acquires a new
	pseudonym (and certificate respectively)

**Table 3:** Simulation parameters and values.

	Parameters	Value
Mobility	Vehicles Number	Simultaneously = 50, 100, 150, 200
		Total = 100, 200, 300, 400
	Insertion method	Quasi-Instant
		(first second insertion)
	Mobility Model	RandomTrips with minimum
		distance = 1500 m
Environment	Used Map	Manhattan grid model
		9 roads, 200 m per segment
	Map size	2000 × 2000 m${}^{2}$
		4 km${}^{2}$
	Simulation Time	300 s
Evaluation	Privacy metrics	Traceability
		N_Traceability
	Pseudonym usage/ consumption	Number of changed-pseudonyms
Strategy	SLOW	Speed threshold = 8 m/s
		Silence threshold = 5 s
	RSP	Pseudonym duration = 60 s
		Silence period = from 3 to 9 s randomly
	CPN	Neighbors radius = 100 m
		Neighbors threshold = 2
	WHISPER	Road neighbors radius = 100 m
		General neighbors radius = 30 m
		Close neighbors radius = 30 m
		Counter default value = 50

**Table 4:** A brief comparison of SLOW, RSP, CPN, and WHISPER strategies according to a set of metrics.

	Staying Silent	Monitoring Neighbors	Pseudonyms Consumption	Safety Ensuring	More Efficiency When
SLOW [16]	✓	✗	Low	✗	Driving in low speeds, hence, keeping silence
RSP [15]	✓	✗	Low	✗	Entering silence and changing pseudonyms synchronously
CPN [14]	✗	✓	Very high	✓	The set of vehicles happens to be large
WHISPER	✗	✓	Medium	✓	Low transmission power condition is satisfied

## Data Availability

The data presented in this study are available on request from the corresponding author. The data are not publicly available due to privacy reasons.
